# Targeted Delivery of ^111^In Into the Nuclei of EGFR Overexpressing Cells *via* Modular Nanotransporters With Anti-EGFR Affibody

**DOI:** 10.3389/fphar.2020.00176

**Published:** 2020-03-04

**Authors:** Tatiana S. Karyagina, Alexey V. Ulasov, Tatiana A. Slastnikova, Andrey A. Rosenkranz, Tatiana N. Lupanova, Yuri V. Khramtsov, Georgii P. Georgiev, Alexander S. Sobolev

**Affiliations:** ^1^Laboratory of Molecular Genetics of Intracellular Transport, Institute of Gene Biology, Russian Academy of Sciences, Moscow, Russia; ^2^Department of Biophysics, Faculty of Biology, Lomonosov Moscow State University, Moscow, Russia

**Keywords:** affibody, cancer, epidermal growth factor receptors, modular nanotransporters, nuclear targeting, targeted drug delivery

## Abstract

Since cell nucleus is one of the most vulnerable compartments, the maximum therapeutic effect from a variety of locally acting agents, such as photosensitizers, alfa-emitters, Auger electron emitters, will be expected when they get there. Therefore, the targeted delivery of these agents into the nuclei of target tumor cells is necessary for their anticancer effects and minimization of side effects. Modular nanotransporters (MNT) are artificial polypeptides comprising several predefined modules that recognize target cell, launching their subsequent internalization, escape from endosomes, and transport the drug load to the nucleus. This technology significantly enhances the cytotoxicity of locally acting drugs *in vitro* and *in vivo*. Epidermal growth factor receptors (EGFR) are useful molecular targets as they are overexpressed in glioblastoma, head-and-neck cancer, bladder cancer, and other malignancies. Here, we examined the possibility of using internalizable anti-EGFR affibody as an EGFR-targeting MNT module for drug transport into the cancer cell nuclei. It binds to both murine and human EGFR facilitating preclinical studies. We showed that MNT with affibody on the N-terminus (MNT_N-affibody_) effectively delivered the Auger electron emitter ^111^In to target cell nuclei and had pronounced cytotoxic efficacy against EGFR-overexpressing human A431 epidermoid carcinoma cells. Using EGFR-expressing human adenocarcinoma MCF-7 cells, we demonstrated that in contrast to MNT with N-terminal epidermal growth factor (EGF), MNT_N-affibody_ and MNT with EGF on the C-terminus did not stimulate cancer cell proliferation.

## Introduction

Today a great interest in pharmacology sphere is the design of drugs that have a local damaging effect and are able to selectively uptake by the target cells (Rosenblum et al., 2018; Sobolev, 2018). Locally acting cytotoxic agents, in particular Auger electron emitters, have great therapeutic potential that can be used to antitumor treatment. These agents are able to damage biomolecules within a few tens of nanometers from their location but have no effect at large distances. Different cell compartments have different resistance to certain damaging agents; the cell nucleus is the most vulnerable compartments to many of them. It determines the importance of development targeted systems for their delivery to reach a significant effect without damaging non-target cells. This approach would minimize side effects and increase the effectiveness of treatment with reducing the minimum required concentration of the drug. Research in this area, in particular, is aimed at creating different intranuclear delivery systems conjugated with locally acting cytotoxic compounds (such as Auger electron emitters, photosensitizers, etc.) (Pan et al., 2018). One such approach is the creation of conjugates with EGF, one of the natural ligands to EGFR (Reilly et al., 2000; Cornelissen et al., 2013).

EGFR, also known as HER1 and ErbB1, has received much attention as a marker, growth driver, and therapeutic target for cancers (Nicholson et al., 2001; Ciardiello and Tortora, 2008; Xu et al., 2017; Sigismund et al., 2018). EGF and several other natural ligands activate EGFR and its downstream signaling pathways (Wang, 2017). Induction of these pro-oncogenic pathways stimulates cancer cell proliferation, migration, survival, DNA-double strand break repair, and hypoxia tolerance, mediates resistance to therapy and inhibits apoptosis (Rodemann et al., 2007; Bussink et al., 2008, Sigismund et al., 2018). Upregulation of this receptor may cause malignization as EGFR plays an important role in the regulation of cell division (Wang, 2017). Abnormal EGFR overexpression and signaling are associated with malignant tumors of the lung (Hirsch et al., 2009), pancreas (Lemoine et al., 1992; Ueda et al., 2004), brain (Hicks et al., 2006), bladder (Chow et al., 1997; Hashmi et al., 2018), breast (Wang et al., 2017), prostate (Peraldo-Neia et al., 2011), and other cancers (Mendelsohn and Baselga, 2000; Ciardiello and Tortora, 2008). EGFR, upon binding to its ligand, is not only capable to activate other signaling proteins, but is also transported inside endosomes from the surface of the cell membrane (Sorkin and Goh, 2009). It has been shown for tumor cells that part of ligand-bound EGF translocates into the cell nuclei (Reilly et al., 2000; Hicks et al., 2006; De Angelis Campos et al., 2011; Sobolev, 2018). Based on the data obtained, numerous attempts have been made to create a drug where EGFR would be used as a vehicle for various agents, such as, for example, Auger-electron emitter ^111^In, Staphylococcal enterotoxin A, etc. Several therapeutic approaches exploiting EGFR for this purpose have been evaluated *in vitro* and *in vivo* (Chen et al., 2002; Song et al., 2016; Zahaf et al., 2017; Liu et al., 2018).

However, since the part of the EGF transported to the nucleus is extremely small (about 7–8%) compared to the cell-bound EGF within 4 h after adding (Reilly et al., 2000), another method should be developed to increase the efficiency of delivery of the cytotoxic agent to the cell nuclei. One of these developments is the modular nanotransporters (MNT) designed in our laboratory (Gilyazova et al., 2006; Rosenkranz et al., 2018). MNT were designed to deliver locally acting drugs such as photosensitizers and radionuclide-emitting short-range particles to the nuclei of the target cells (Sobolev, 2008; Sobolev et al., 2016; Sobolev, 2018). We used Auger electron emitters, because they combine two important characteristics. On the one hand, Auger electrons have a high linear energy transfer, which leads to multiple damage of macromolecules, and on the other hand, their path length is extremely small and in most cases does not exceed several tens of nanometers, which greatly reduces the cytotoxicity of Auger electron emitters for tissues if decay occurs outside the cell nucleus (Kassis and Adelstein, 2005).

**Figure 1:**
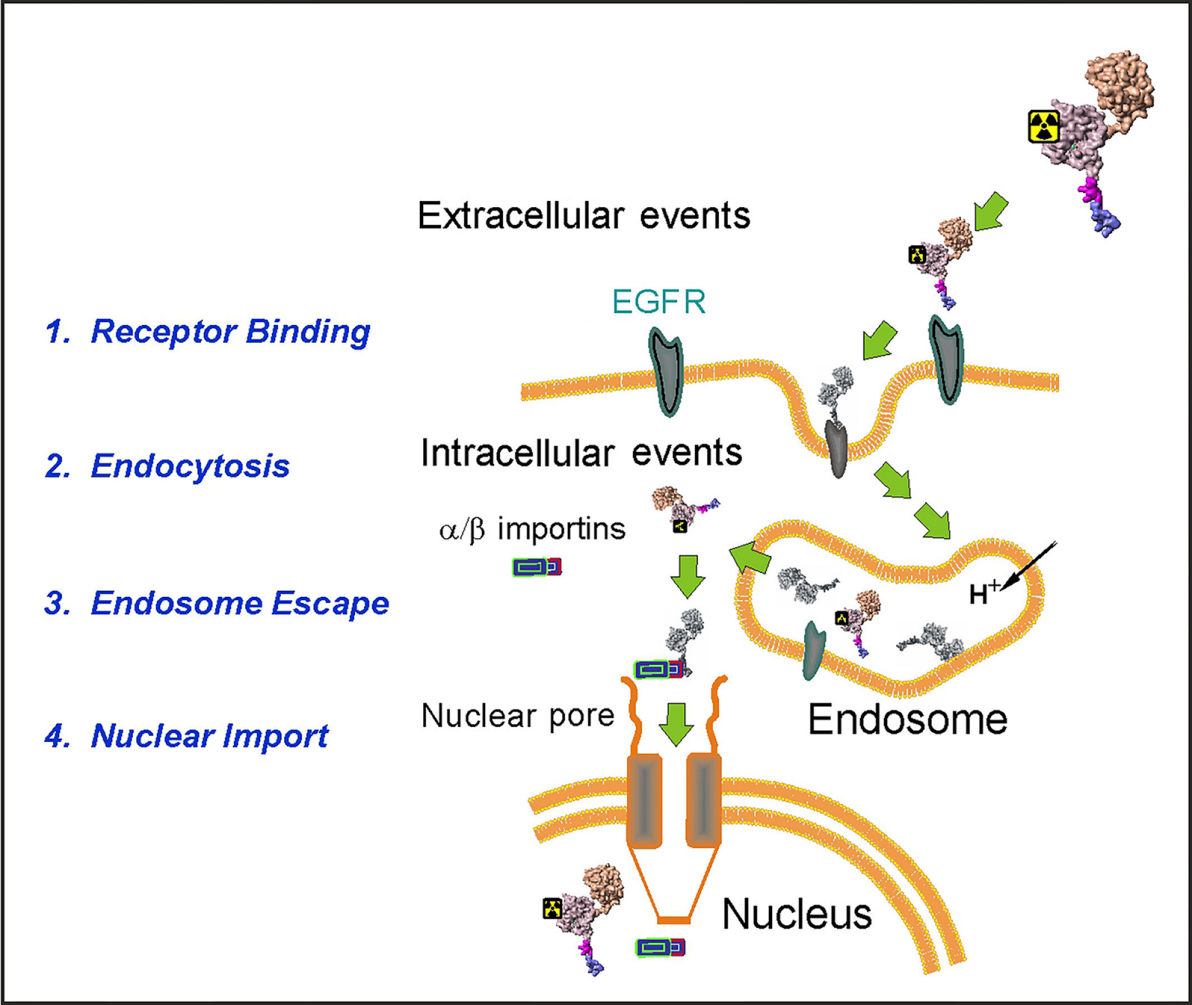
A schematic diagram depicting the stages of the MNT transport to the cell nucleus of the EGFR-expressing target cells. (Reproduced with slight modification from Sobolev, 2008 Bioessays. 2008 Mar;30(3):278-87. doi: 10.1002/bies.20715, with permission).

We designed an EGFR-recognizing modular nanotransporter consisting of EGF as a ligand module for selective recognition of target cells overexpressing EGFR, an endosomolytic module based on the translocation domain of the diphtheria toxin, a module containing an optimized nuclear localization sequence (NLS) of the SV40 large T antigen for active nuclear transport by the importin-α/β carrier protein complex (Goldfarb et al., 2004), and a carrier module based on the *E. coli* hemoglobin-like protein HMP (Rosenkranz et al., 2008). Scheme of the MNT transport into the cell nucleus of a target cell is depicted in **Figure 1**.

This modular nanotransporter is named DTox-HMP-NLS-EGF (or, MNT_C-EGF_, where C-EGF indicates that EGF is localized to the *C*-terminal in the MNT). The experiments with this MNT were carried out on several cell lines in particular on human A431 epidermoid carcinoma cells. It was shown that MNT_C-EGF_ efficiently delivered drugs to the cell nucleus of the target cells and enhanced their cytotoxic efficacy *in vitro* (Gilyazova et al., 2006; Rosenkranz et al., 2008; Slastnikova et al., 2012a; Koumarianou et al., 2014; Slastnikova et al., 2017b; Rosenkranz et al., 2018) and in vivo (Slastnikova et al., 2012a; Slastnikova et al., 2012b; Slastnikova et al., 2017b; Rosenkranz et al., 2018). Thus, in *in vitro* experiments it was shown that the concentration of the drug agent (Auger electron emitters ^111^In, ^67^Ga, ^125^I, or alpha-particle emitter ^211^At or photosensitizers bacteriochlorin p or chlorin e_6_) corresponding to 50% survival of cancer cells was up to 3000 times higher for free chlorin e_6_ or corresponding to 37% survival up to 4000 times for ^125^I compared to MNT-agent conjugates (Gilyazova et al., 2006; Rosenkranz et al., 2008; Slastnikova et al., 2012a; Koumarianou et al., 2014; Slastnikova et al., 2017b). *In vivo* experiments on tumor-bearing mice proved that locoregional injection of MNT_C-EGF_ conjugated with ^111^In resulted in significant tumor growth inhibition compared to tumor-bearing animals receiving a corresponding dose of non-labeled MNT or free ^111^In (Rosenkranz et al., 2018).

When EGF is used as a ligand in drug delivery vehicles, it can affect cellular signaling and stimulates events leading to enhances cancer cell proliferation (Chen et al., 2002; Kim et al., 2015). EGF and other natural ligands induce dimerization and autophosphorylation of EGFR (Schlessinger, 2002) and subsequent internalization (Friedman and Stahl, 2009) of the receptor. Downstream activation of the phosphatidylinositol-3-kinase (PI3-K)/protein kinase B (AKT) (PI3-K/AKT pathway), Ras and STAT signaling cascades (Citri and Yarden, 2006; Rodemann et al., 2007) leads to stimulation of proliferation, inhibit apoptosis, and promote migration (Seshacharyulu et al., 2012). In order to avoid ligand-mediated activation of the downstream cascades researchers developed alternative EGFR ligands such as EGFt, a truncated form of human EGF (hEGF) lacking the eight *C*-terminal amino acids (Panosa et al., 2015), artificial EGFR ligands such as small antibody-like protein on the basis of Z-domain of A-protein: anti-EGFR affibody (Z_EGFR_:1907) (Kim et al., 2012; Stahl et al., 2017), and anti-EGFR nanobody (Roovers et al., 2007).

In our previous experiments we used MNT with EGF at the C-terminus (MNT_C-EGF_) and revealed that it had no effect on cell growth (Rosenkranz et al., 2018). However, as *in vivo* degradation of the EGF-containing MNT potentially can lead to EGFR-activating products, we examined the possibility of using a new MNT with the affibody to EGFR. Affibody Z_1907_ does not influence on cell proliferation (Ekerljung et al., 2012), so we tested it as a ligand module. Based on the findings of earlier studies (Kim et al., 2012; Kim et al., 2013), we placed the affibody on the *N*-terminus of the MNT. Therefore, another reason of creating the new MNT was a palette extension, which would expand the possibility of modification of the MNT with additional functional modules. MNT_C-EGF_ and the new MNT with EGF at the *N*-terminus (MNT_N-EGF_) served as controls. The effects of these MNT on the proliferation of cells overexpressing surface EGFR were investigated. We also explored the ability of these MNT to deliver the cytotoxic agent ^111^In, an Auger-electron emitter, to target cell nuclei.

## Materials and Methods

### Materials

Human A431 epidermoid carcinoma and MCF-7 adenocarcinoma cells were obtained from the American Type Culture Collection (ATCC; Manassas, VA, USA). They were passaged in the laboratory less than 30 times. All media, cell culture components, and 3-[4,5-dimethylthiazol-2-yl]-2,5 diphenyltetrazolium bromide (MTT) were purchased from Paneco (Moscow, Russia). *Escherichia сoli* C3029 cells were purchased from New England Biolabs (Ipswich, MA, USA). Ni-NTA agarose was purchased from Qiagen (Hilden, Germany). Alexa Fluor 647 succinimidyl ester was purchased from Molecular Probes (Eugene, OR, USA). The bifunctional chelator 2-*S*-(4-isothiocyanatobenzyl)-1,4,7-triazacyclononane-1,4,7-triacetic acid (p-SCN-BnNOTA) was obtained from Macrocyclics (Plano, TX, USA). The ^111^InCl_3_ was obtained from Zavod Medradiopreparat (Moscow, Russia). The ^125^I was acquired from the Khlopin Radium Institute (Saint Petersburg, Russia). The 1,4-dithiothreitol (DTT) was purchased from Fisher Scientific (Schwerte, Germany). Mini-Protean^®^ TGX Any kD™ gels were purchased from Bio-Rad Laboratories (Hercules, CA, USA). LB Broth Miller-Novagen, a granulated *E. coli* cultivation medium, was purchased from Helicon (Moscow, Russia). EGTA and EDTA were obtained from Serva (Heidelberg, Germany). Isopropyl β-d-1-thiogalactopyranoside (IPTG) and Amicon Ultracel-30K centrifugal filter units were procured from Merck (Darmstadt, Germany). Antibodies were purchased from Abcam (Cambridge, UK). SYBR Green was purchased from Lumiprobe (Moscow, Russia). 1,3,4,6-Tetrachloro-3α,6α-diphenylglycouril (Iodogen), KCl, NaHCO_3_, MgCl_2_, and CaCl_2_ were purchased from Sigma-Aldrich (St. Louis, MO, USA). Calcein was obtained from Fluka (Munich, Germany).

### Methods

#### Cell Culture

Human A431 epidermoid carcinoma and MCF-7 adenocarcinoma cells overexpressing EGFR were maintained in Dulbecco’s modified Eagle’s medium (DMEM) supplemented with 10% calf fetal serum (CFS) and 50 μg ml^−1^ gentamicin at 37°C under a 5% CO_2_ humidified atmosphere.

#### Plasmid Construction and Protein Purification

A DNA fragment encoding EGF was amplified by PCR from the 6His-DTox-HMP-NLS-EGF (MNT_C-EGF_) gene and subcloned into a plasmid encoding 6His-DTox-HMP-NLS (Gilyazova et al., 2006). The product was EGF-6His-DTox-HMP-NLS (MNT_N-EGF_). The anti-EGFR affibody gene was synthesized by General Biosystems (Morrisville, NC, USA) based on a published amino acid sequence (Stahl et al., 2017), and subcloned into 6His-DTox-HMP-NLS. The product was the affibody-6His-DTox-HMP-NLS (MNT_N-affibody_) plasmid.

*E. coli* C3029 cells were transformed with MNT_C-EGF_, MNT_N-EGF_, or MNT_N-affibody_ plasmids and grown on LB Broth Miller-Novagen with ampicillin (100 μg ml^−1^) to A_600_ = 0.6 at 37°C. They were induced overnight with 0.2 mM IPTG at 18°C. Proteins were purified with Ni-NTA from the soluble fraction of *E. coli* lysate as previously described (Gilyazova et al., 2006).

#### Binding of the MNT to the EGFR

Binding of the new MNT to EGFR was assessed on A431 cells by a competitive radioligand binding assay with ^125^I-labeled EGF as previously described using Iodogen (Rosenkranz et al., 2008; Slastnikova et al., 2012a).

EGF and 40 MBq of radioiodide in 0.05 M sodium borate buffer (pH 8.5) were incubated in glass vials coated with 10 μg of Iodogen for 15 min on ice. Radioiodinated EGF was purified by gel-filtration through a PD-10 column (GE Healthcare, Chicago, IL, USA) that was eluted with phosphate-buffered saline (pH 7.5).

The competitive radioligand analysis MNT binding was accomplished with A431 cells in 48-well plates overnight at 4°C with 1 nM of ^125^I-EGF and indicated concentrations of MNT in the DMEM medium without sodium bicarbonate supplemented with 10 mg/ml of bovine serum albumin and 20 mM 4-(2-hydroxyethyl)-1-piperazineethanesulfonic acid (pH 7.5). The cells were washed four times with the same medium on ice, lysed in 1 M NaOH for 30 min, and the radioactivity associated with the cell lysates was measured using RiaGamma counter 1271 (LKB Wallac, Sollentuna, Sweden).

#### Functional Activity of the Endosomolytic Module

The ability of the new MNT to provide liposome leakage was demonstrated on unilamellar liposomes from egg lecithin (Khimpharmzavod, Kharkov, Ukraine) loaded with the fluorescent dye calcein (Fluka, Munich, Germany) in accordance with a previously published protocol (Khramtsov et al., 2008). In brief, the unilamellar liposomes loaded with fluorescent calcein up to the concentration of fluorescence quenching (100 mM) were prepared by sonicating fresh lipid suspension in 20 mM HEPES, 20 mM MES, 20 mM citrate, 150 mM NaCl, pH 7.4 (liposome buffer) until clear, using a W-181-T sonicator (Finnsonik, Lahti, Finland; 40 kHz, 90 W, 0°C, 30 min), and passed 10 times through Durapore filters with 0.22 μm pore diameter (Millipore, Burlington, MA, USA) to standardize liposomes sizes. The liposomes were stored under an argon atmosphere at 4°C for several months. PD-10-purified liposomes were incubated with 100 nM MNT for 30 min in liposome buffer at indicated pH (3–7.5) in triplicates after that samples were diluted tenfold in liposome buffer, pH 7.5 and fluorescence of leaked calcein was measured at 520 nm at excitation wavelength 490 nm. As a positive control (100% calcein leakage) we used addition Triton X-100 up to 0.5%. The samples without MNT were used as background leakage.

#### Conjugation of Alexa Fluor 647 to MNT

Freshly prepared 9.8 mM Alexa Fluor 647 succinimidyl ester was added in 5:1 molar excess to MNT_N-EGF_, MNT_N-affibody_, MNT_C-EGF_, or ligand-free MNT solutions in carbonate buffer at pH 8.6. After overnight incubation with gentle stirring at 4°C, the Alexa Fluor 647-labeled MNT were separated from the unreacted fluorophore by five cycles of Amicon Ultracel-30K ultrafiltration. The quantification of Alexa Fluor 647 MNT labeling (near 3.5 Alexa residues per MNT for all of them) was carried out by spectrophotometry. Alexa Fluor 647 extinction coefficient ϵ_650nm_ = 270,000 M^–1^ cm^–1^. The MNT protein concentration was determined by the Bradford assay.

#### Flow Cytometry Studies of Alexa Fluor 647-labeled MNT Internalization in EGFR-expressing Cells

EGFR-expressing A431 cells were seeded in 24-well plates (2.5 × 10^4^ cells/well), a couple days afterward the medium was changed for a fresh one and AlexaFluor 647 labeled MNT were added (n = 4 per each point) to a final concentration of 100 nM. After 18-h incubation at 37°C in a 5% CO^2^ humidified atmosphere, medium containing unbound MNT was removed and the cells were washed thrice, trypsinized to detach and remove cell-surface bound MNT, harvested, dissolved in Hanks’ solution with CFS and analyzed by flow cytometry using an Epics Altra Flow Cytometer (Beckman Coulter, Miami, Florida, USA). The Alexa Fluor 647 dye was excited at 633 nm, and emission was detected at 675 nm. A total of 1 × 10^4^ gated events were collected per sample. To assess the nonspecific uptake parallel wells (n = 4 per each point) with free EGF excess (2 μM) were processed in the same way. Untreated cells were used as an autofluorescence control.

#### Confocal Laser-Scanning Microscopy Imaging

EGFR-expressing A431 cells were seeded in 24-well cell imaging black plates with glass bottoms (1.5 × 10^4^ cells well^−1^). After 2 d, the medium was changed, and Alexa Fluor 647-labeled MNT were added to final concentrations of 50 nM.

Prior to imaging, SYBR Green (1:10,000 dilution) was added to the cells to visualize the nuclei. After incubation with Alexa Fluor 647-labeled MNT, the cells were examined under the LSM-510 Meta NLO multiphoton laser scanning microscope fitted with a Plan-Apochromat ×63/1.4 Oil DIC lens (Carl Zeiss, Oberkochen, Germany). SYBR Green fluorescence was recorded at an excitation wavelength of 488 nm and an emission wavelength bandpass of 500 to 530 nm. Alexa Fluor 647 fluorescence was recorded at an excitation wavelength of 633 nm and an emission wavelength bandpass of 650 to 710 nm. The mean intranuclear fluorescence at 7 and at 48 h of Alexa 647-labeled MNT toward SYBR Green were calculated using multiphoton laser scanning microscope software.

#### Interactions of the MNT with α/β Importins Evaluated by Thermophoresis

Importins α and β were obtained as previously described (Gilyazova et al., 2006). Importin β was conjugated with Cy3 dye and separated from unbound dye on a PD-10 column (GE Healthcare, Chicago, IL, USA). Importins were diluted in importin buffer (pH 7.4) containing 20 mM HEPES, 110 mM KCl, 5 mM NaHCO_3_, 5 mM MgCl_2_, 0.1 mM CaCl_2_, 1 mM EGTA, and 1 mM DTT. Importin heterodimers were used at equimolar concentrations of 50 nM. MNT_N-EGF_, MNT_c-EGF_, and MNT_N-affibody_ were serially diluted in buffer with α/β importins. The initial concentrations were 1 µM for MNT_N-EGF_ and 4 µM for MNT_N-affibody_ and MNT_C-EGF_. The strengths of the interactions between the MNT and the α/β importins were measured with a MonolithNT.115 instrument (NanoTemper Technologies, Munich, Germany). The binding affinities (K_d_) were automatically interpolated from a fitted curve by MonolithNT.115 Instruments software (NanoTemper Technologies, Munich, Germany).

#### Labeling MNT With ^111^In Using p-SCN-Bn-NOTA

MNT_C-EGF_, MNT_N-affibody_, and MNT_N-EGF_ were labeled in accordance with a previously published protocol (Slastnikova et al., 2017a). MNT were incubated with 10-fold molar excess of the bifunctional p-SCN-Bn-NOTA chelator in conjugation carbonate buffer at pH 8.6 (Hens et al., 2009) for 20 h at room temperature with final concentrations of MNT ≥1.5 mg/ml. The chelator-MNT conjugate was concentrated and separated from excess chelator by five cycles of ultrafiltration using Amicon Ultracel-30K. During this process, the conjugation buffer was gradually replaced with 10 mM 4-(2-hydroxyethyl)-1-piperazineethanesulfonic acid (HEPES), 15 mM NaCl, pH 7.4. All buffers used for chelator conjugation and labeling procedures were passed through Chelex-100 resin (200–400 mesh; Bio-Rad) to minimize adventitious metal ion contamination.

For ^111^In labeling NOTA-MNT_C-EGF_, NOTA-MNT_N-affibody_ and NOTA-MNT_N-EGF_ (0.2 mg, 0.04 mg, and 0.04 mg, respectively) in 10 mM HEPES, 15 mM NaCl, pH 7.5, was mixed with 1 M HEPES, pH 7.5, 0.1 M citrate, pH 6.7, 1% SDS, and 0.25 M HCl (Ultrapure Grade, Merck, Darmstadt, Germany); then ^111^InCl_3_ in 0.048 M HCl was added. The reaction mixture was incubated at 37°C for 1 h, and then the reaction was stopped by adding 3 μl 0.05 M EDTA, pH 8.0, followed by gentle mixing and incubation for 10 min at 37°C. Finally, the pH was neutralized with 1 M NaOH. The initial specific radioactivity of either FR-targeted ^111^In-MNT, obtained using this protocol, was 2.7 GBq mg^−1^. As a control, ^111^In was treated following the same procedures except that the NOTA-MNT was omitted in the reaction mixture. Radiochemical yields and ^111^In-MNT integrity were analyzed by Laemmli SDS-PAGE using Mini-Protean TGX Any kD gels. Radioactivity was detected on a Storm 865 phosphor imager (GE Healthcare, Uppsala, Sweden). Images were analyzed by ImageQuant TL v. 5.0 software (Bio-Rad Laboratories, Hercules, CA, USA).

#### Cytotoxicity Studies

A431 cells were seeded in 24-well plates (2 × 10^4^ cells well^−1^). After 2 d, the media were refreshed and various dilutions of ^111^In-NOTA-MNT (0–6.5 MBq ml^−l^; 0–32 μg ml^−l^) were added. The ^111^In (0–20 MBq mv^−l^) was used as a control. The cells were incubated for 48 h at 37°C under a 5% CO_2_ atmosphere. Medium containing unbound radioactivity was removed. The cells were washed by Versene solution, trypsinized, harvested, and resuspended in 1 ml fresh medium with 10% (w/v) CFS. For a colony-forming assay, the cells were seeded in 25-cm^2^ flasks (2,000 cells flask^−1^) containing DMEM/F12 supplemented with 10% (w/v) CFS. After 8 d, the colonies were stained with crystal violet and counted. Fitting was made in accordance with «one phase decay» algorithm using GraphPad Prism 5 software.

#### ^111^In-NOTA-MNT Accumulation in the Nuclei

A431 cells were seeded in 12-well plates (5 × 10^5^ cells well^−1^) containing DMEM supplemented with 10% CFS. After 2 d, the media were refreshed and various dilutions of ^111^In-NOTA-MNT (0.6 MBq ml^−1^; 1.9 μg ml^−1^) were added. The cells were incubated for 2 h at 37°C under a 5% CO_2_ atmosphere. The plates were cooled and the media containing unbound radioactivity were removed. The cells were washed by Versene solution, trypsinized, and harvested. The wells were washed with ice-cold DMEM containing 10% (w/v) CFS, and the rinsate was added to the cells samples. The cells were then centrifuged in an Eppendorf centrifuge 5415R at 200 × *g* and 4°C for 7 min, resuspended in 500 μl cold fresh medium with 10% (w/v) CFS, and re-centrifuged. The supernatant was removed, and the cells were resuspended in 300 μl ice-cold hypotonic buffer (25 mM Tris-HCl (pH 7.5), 5 mM KCl, 0.5 mM DTT, 1 mM PMSF, and 0.15 U ml^−1^ aprotinin) and expanded on ice for 20 min. The mixture was homogenized on ice with a Dounce homogenizer (15 strokes). Nuclei were pelleted by centrifugation at 600 × *g* and 4°C for 12 min. Pelleted nuclei were washed 5× in 300 μl·wash^−1^·tube^−1^ ice-cold isotonic buffer (0.25 M sucrose, 6 mM MgCl_2_, 10 mM Tris-HCl (pH 7.4) 0.5% (w/v) Triton X-100, 1 mM PMSF, and 0.15 U ml^−1^ aprotinin) to remove cytoplasmic membranes.

Nuclear purity was confirmed by microscopic evaluation. The nuclei were resuspended in a Versene solution and incubated on ice for 15 to 30 min to reduce clumping. The suspension was centrifuged at 600 × *g* and 4°C for 12 min, and the nuclear yield was determined using counting chambers.

Sample radioactivity was measured with a RiaGamma counter 1271 (LKB Wallac, Sollentuna, Sweden). The percentages of activity in the nuclei relative to the total intracellular activity were calculated for all ^111^In-NOTA-MNT.

Nuclear purity was evaluated by western blot using antibodies against α-tubulin (a cytoplasmic marker) and nibrin (NBS-1; a nuclear marker).

#### Cell Proliferation

MCF-7 cells were seeded in 24-well plates (8 × 10^3^ cells well^−1^) at day 0 in DMEM supplemented with 0.5% (w/v) CFS. After 1 d, the medium was refreshed with 2 ml DMEM and 0.5% CFS per well. MNT_C-EGF_, MNT_N-affibody_, MNT_N-EGF_, MNT without ligand module (MNT_w/l_), and human EGF were added to the cells to a final concentration of 100 nM. The media were not changed during the experiment. Cell growth at the indicated time points was estimated using 0.2 ml of MTT. The resultant formazan crystals were solubilized in 2 ml of 96° ethanol:DMSO (1:1) per well. Absorbances and the background were read at 570 and 650 nm, respectively, on a Biotek Synergy 4 microplate reader (Winooski, VT, USA).

#### Statistics

The data were analyzed using GraphPad Prism 5 software (GraphPad Software Inc., San Diego, CA, USA). Data on the plots represent mean values, with bars indicating the standard error of the mean of repetitive values. The significance of the difference was evaluated using the Mann–Whitney U-test or Tukey multiple comparison test. The differences were significant when P < 0.05.

## Results

**Figure 2:**
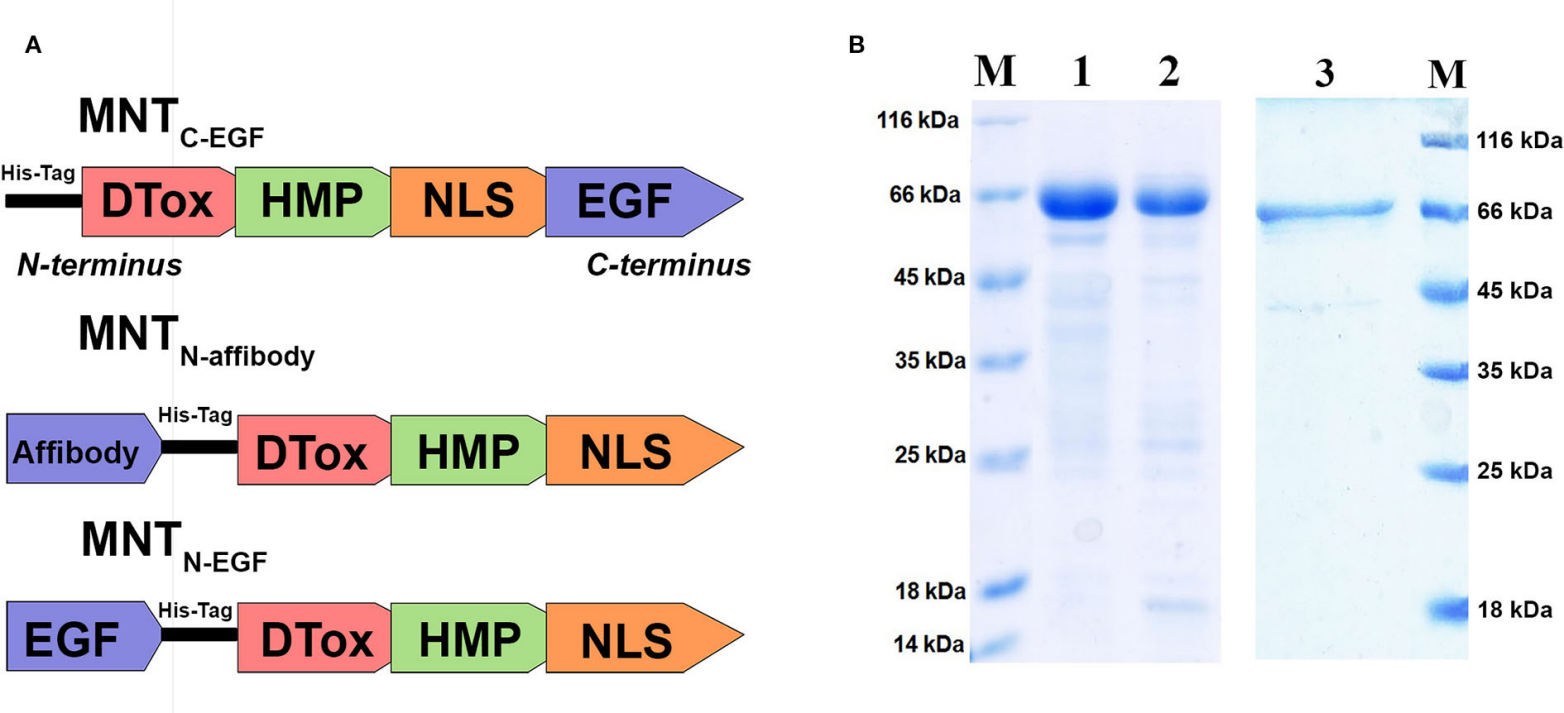
Characteristics of the MNT. **(A)** A schematic diagram depicting structure of the new MNT_N-affibody_ and MNT_N-EGF_ and previously described MNT_C-EGF_. **(B)** SDS-PAGE gels of MNT_N-affibody_ (lane 1), MNT_N-EGF_ (lane 2), MNT_C-EGF_ (lane 3) and unstained protein markers (lane M).

Here, we produced and characterized MNT_N-affibody_, the new EGFR-binding MNT. Within the previously created and characterized MNT_C-EGF_ (Gilyazova et al., 2006), we transferred its ligand module from C-terminus to N-terminus and replaced EGF with the Z_1907_ affibody (Kim et al., 2012). MNT_N-EGF_ was also derived from the MNT_C-EGF_ (**Figure 2A**). The sequences of these MNT are shown in **Supplements 1**.

Purity of isolated and purified MNT_N-affibody,_ MNT_C-EGF_ and MNT_N-EGF_ was evaluated by Laemmli SDS PAGE, it was > 85% for all MNT. SDS-PAGE gel of MNT_N-affibody_ and MNT_N-EGF_ was shown in **Figure 2B**.

**Figure 3:**
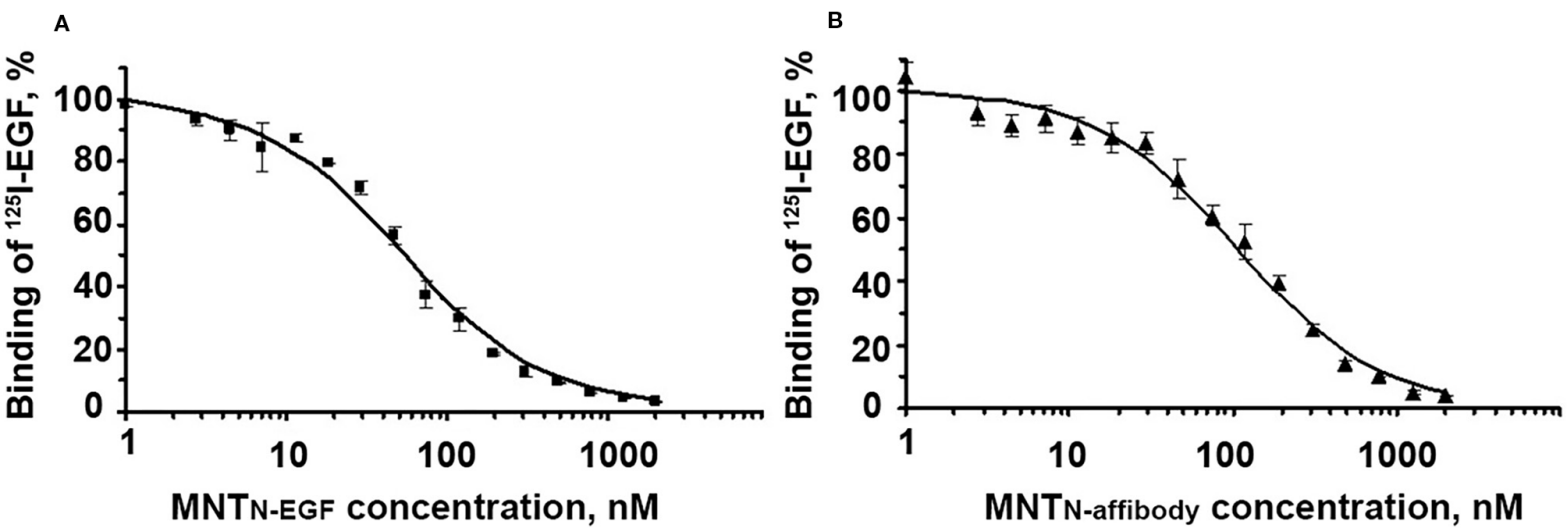
Displacement curves for [^125^I]iodo-EGF by MNT_N-EGF_ **(A)** and MNT_N-affibody_ **(B)** from EGFR on A431 cells. [^125^I]iodoEGF (1 nM) and MNT at the indicated concentrations were added to the cells and incubated at 4°C for 20 h. Error bars represent standard errors of the mean (SEM; n = 3).

The binding of MNT to EGFR was evaluated with an EGFR-expressing human A431 epidermoid carcinoma cell line. The dissociation constants for MNT_N-EGF_ and MNT_N-affibody_ were interpolated from displacement curves (**Figure 3**) and were 37.5 ± 5.9 nM and 34.7 ± 4.1 nM, respectively. These values are close to those of the prototypical MNT_C-EGF_ (29.3 nM) (Rosenkranz et al., 2008) and indicate the ability of the new MNT to specifically bind to the EGFR, that is necessary for receptor-specific recognition of the target cells.

**Figure 4:**
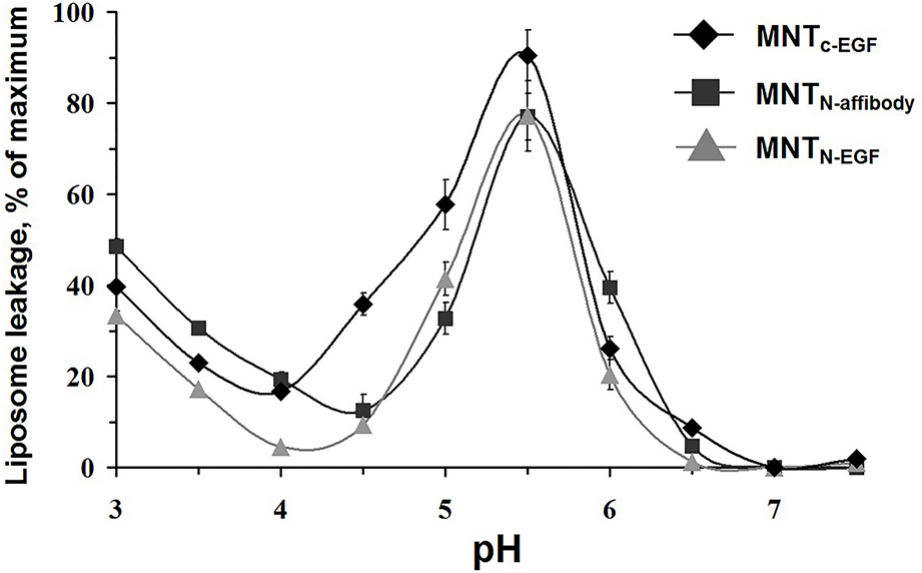
MNT_C-EGF_, MNT_N-affibody_, and MNT_N-EGF_ induced leakage from egg yolk phosphatidyl choline liposomes loaded with fluorescent calcein to the fluorescence quenching concentration. The appearance of fluorescence indicates liposome leakage. Error bars are SEM (n = 4).

The propensity of MNT to make membrane pores *via* the endosomolytic module was tested using calcein-loaded liposomes at various pH. Maximum pH-dependent calcein leakage (**Figure 4**) was observed at pH = 5.5 for all three assessed MNT. This weakly acidic internal pH corresponds to that of the endosome (Canton and Battaglia, 2012). Accordingly, relocation and change of the ligand module do not influence endosomolytic activity of the DTox module, and the new MNT are potentially able to escape from endosomes before fusion of late endosomes with lysosomes and subsequent lysosomal degradation.

**Figure 5:**
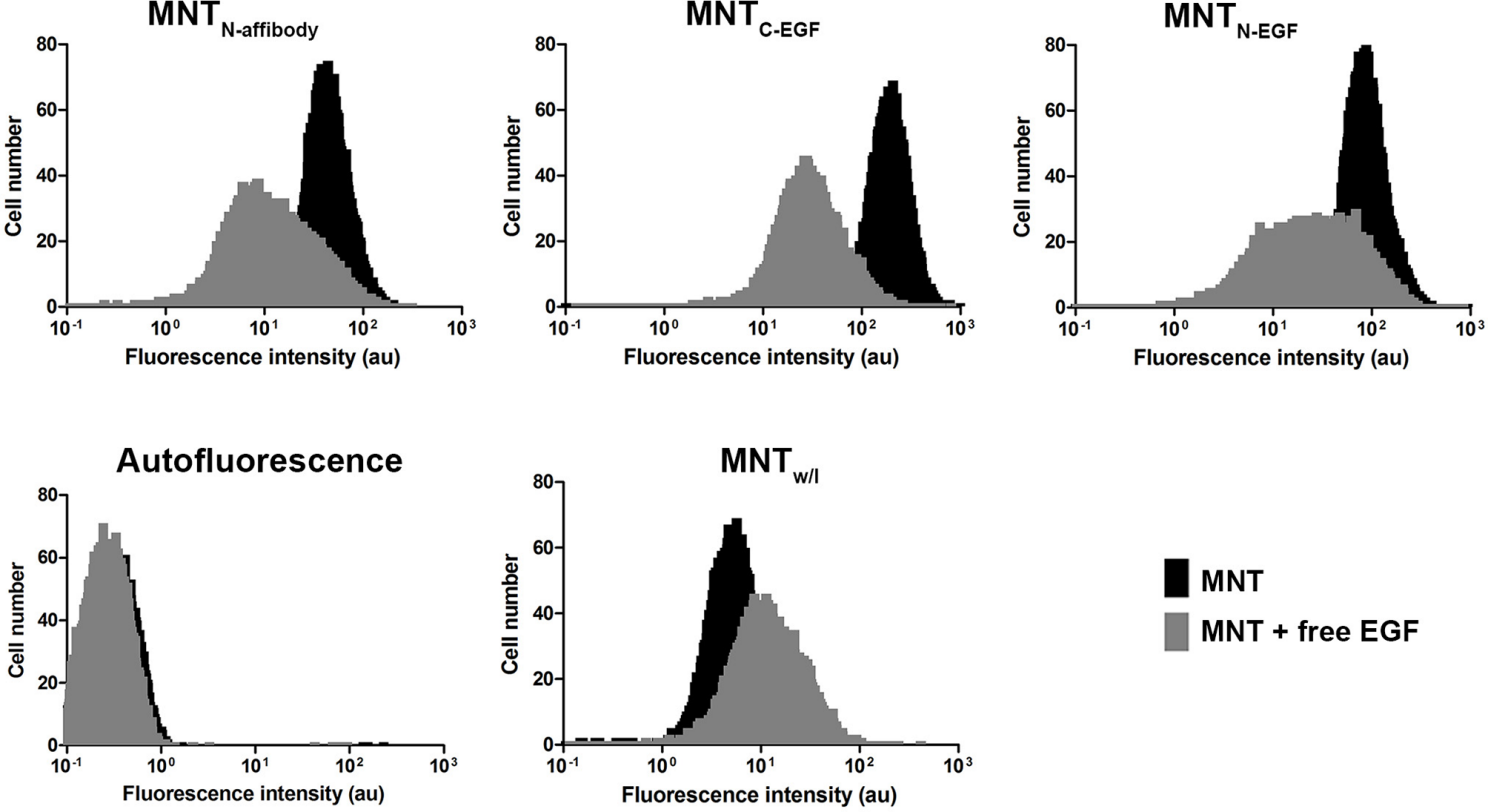
Intracellular accumulation of Alexa 647 labeled MNT measured by flow cytometry. Flow cytometry histograms of A431 cells, cells incubated with MNT_N-affibody_, MNT_N-EGF_, MNT_C-EGF_ or with control MNT_w/l_ for 18 h with or without free EGF (2 μM). Untreated cells served as autofluorescence controls. Error bars represent standard errors of mean (n = 4).

The ability of the Alexa Fluor 647 labeled MNT to internalize into the target EGFR-expressing cells was shown using the flow cytometry method. Since the cells incubated with MNT were washed and trypsinized, the detected fluorescence signal corresponded to internalized part of the MNT. For the cells incubated with EGFR-binding MNT, fluorescence intensity value corresponding to the maximum of cell amount significantly differs from fluorescence intensity value for non-incubated cells (autofluorescence). The smaller difference in the signal for MNT_w/l_ from autofluorescence indicates that MNT_w/l_ transport into the cell was significantly worse than that with MNT_N-affibody_, MNT_N-EGF_, and MNT_C-EGF_ (**Figure 5**). Addition of 2 μM free EGF to the medium significantly reduced the average fluorescence intensity for EGFR-binding MNT that indicates the importance of the contribution of receptor-mediated transport. The significance of the difference was evaluated using the Mann–Whitney U-test.

**Figure 6:**
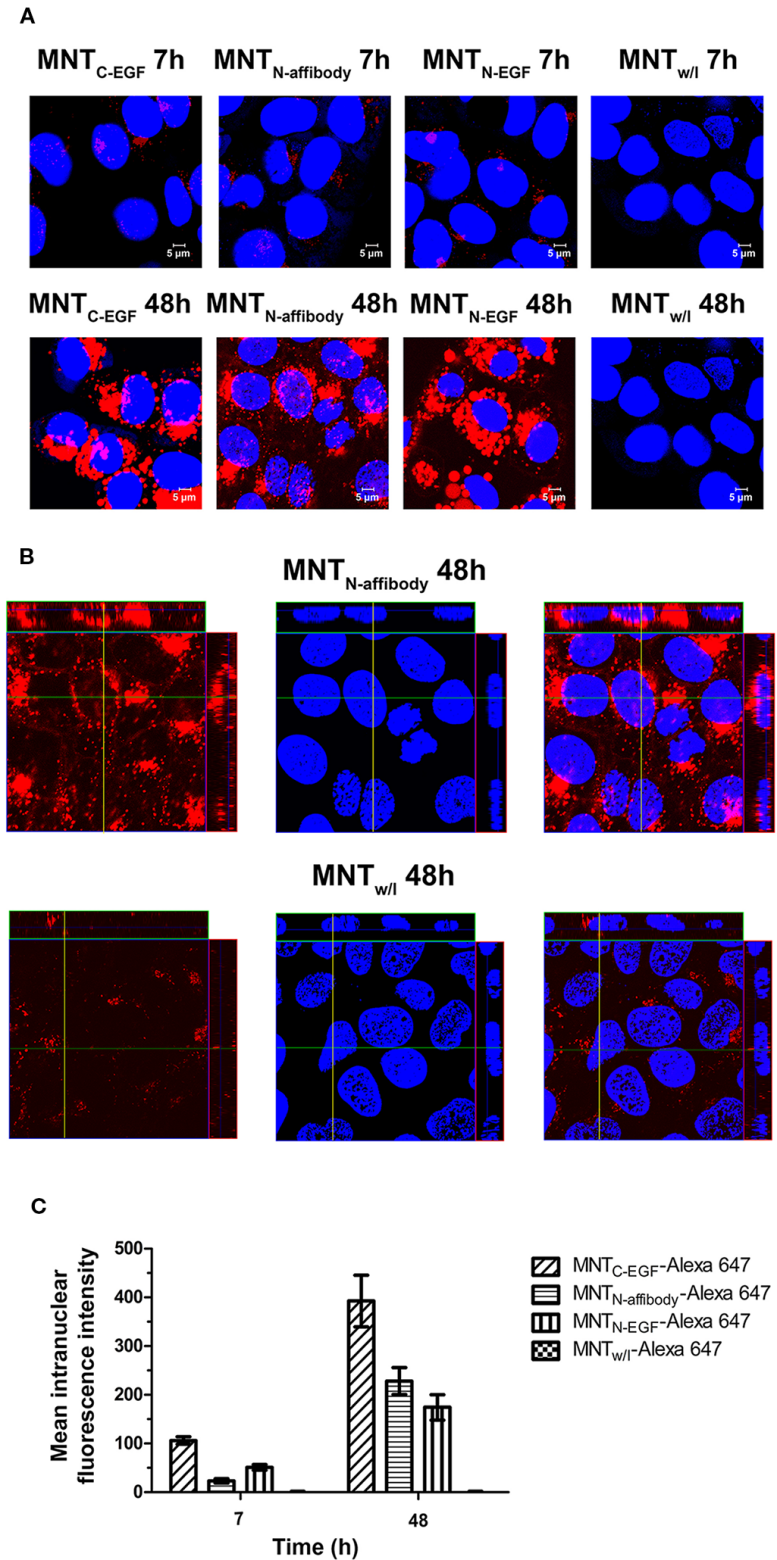
Intranuclear distribution of MNT_C-EGF_, MNT_N-affibody_, MNT_N-EGF_, and MNT without ligand module (MNT_w/l_), labeled with Alexa Fluor 647, and visualized by confocal laser scanning microscopy inside A431 cells. **(A)** Confocal laser scanning microscopy images across the nuclei of A431 cells incubated for 7 and 48 h with 50 nM Alexa 647-labeled MNT. Alexa Fluor 647 fluorescence corresponding to MNT is in red pseudo-color. Nuclei were counterstained with SYBR Green (blue pseudo-color). **(B)** Confocal laser scanning microscopy images across the nuclei of A431 cells incubated for 48 h with 50 nM Alexa 647-labeled MNT_N-affibody_ and with MNT_w/l_ as a control. Alexa Fluor 647 fluorescence corresponding to MNT is in red pseudo-color. Nuclei were counterstained with SYBR Green (blue pseudo-color). **(C)** Mean intranuclear fluorescence intensity of Alexa 647-labeled MNT following 7 and 48 h incubation. Error bars are SEM (n = 90–134).

The intranuclear localizations of MNT labeled with Alexa Fluor 647 were viewed in A431 cells under confocal laser scanning microscopy. For all types of MNT with EGFR ligand modules, the Alexa Fluor 647 signal was visible within the nuclei from 7–48 h incubation. For MNT_w/l_, there was no substantial signal in the nuclei (**Figures 6A, B**). The intranuclear signal intensity increased from 7 h to 48 h for all EGFR-binding MNT (**Figure 6C**). The data obtained indicate the ability of new MNT to transport into the nuclei of the target cells and accumulate there in process of time.

**Figure 7:**
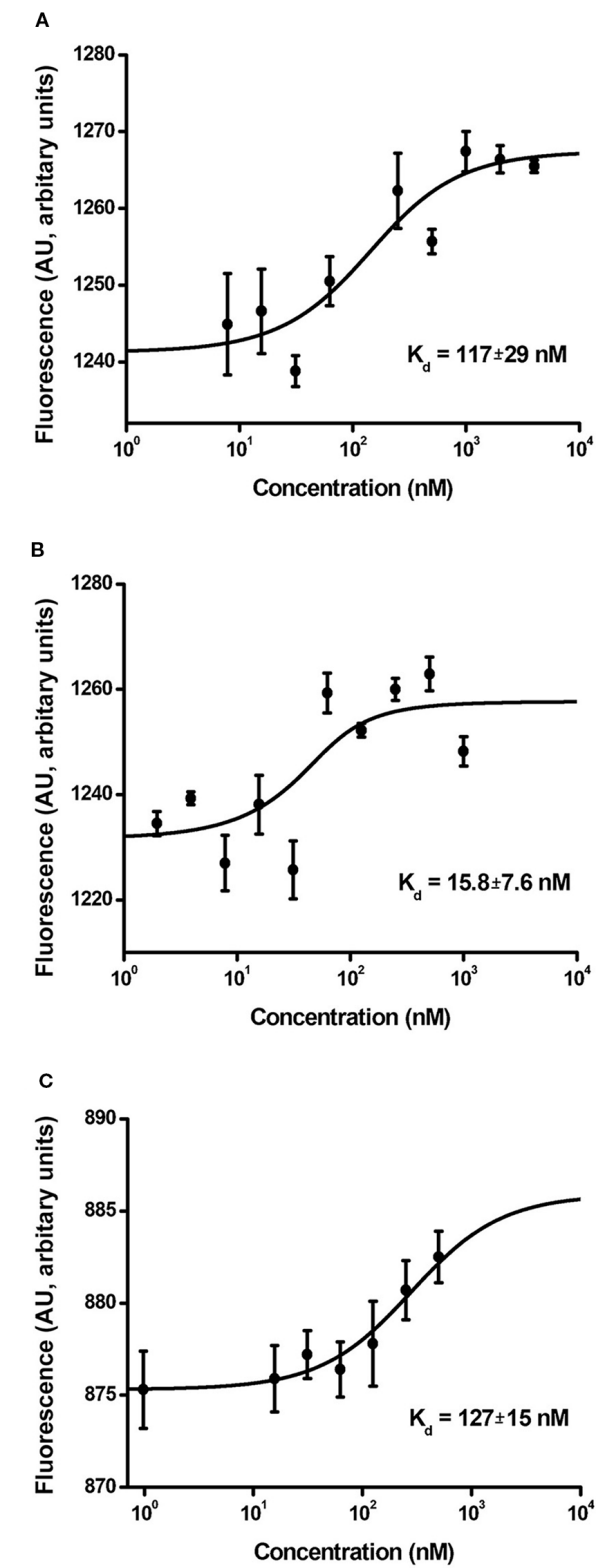
Interaction curves of MNT_N-affibody_ **(A)**, MNT_N-EGF_ **(B)**, and MNT_C-EGF_ **(C)** with α/β importins. Binding of serial MNT dilutions with α/β importin heterodimer was measured by thermophoresis. Binding affinity (K_d_) was automatically interpolated from a fitted curve by MonolithNT.115 Instruments software. Error bars are SEM (n = 3–9).

The binding affinity of MNT for the importin-α/β carrier protein complex reflects the functional activity of the NLS module responsible for nuclear MNT import. The dissociation constant (K_d_) for MNT_N-affibody_ binding to the α/β importin heterodimer is 117 ± 29 nM (**Figure 7A**). It was automatically calculated from the interaction thermophoresis curve and closely approaches the value for MNT_C-EGF_ (127 ± 15 nM; **Figure 7C**). In contrast, the value for MNT_N-EGF_ is 15.8 ± 7.6 nM (**Figure 7B**).

**Figure 8:**
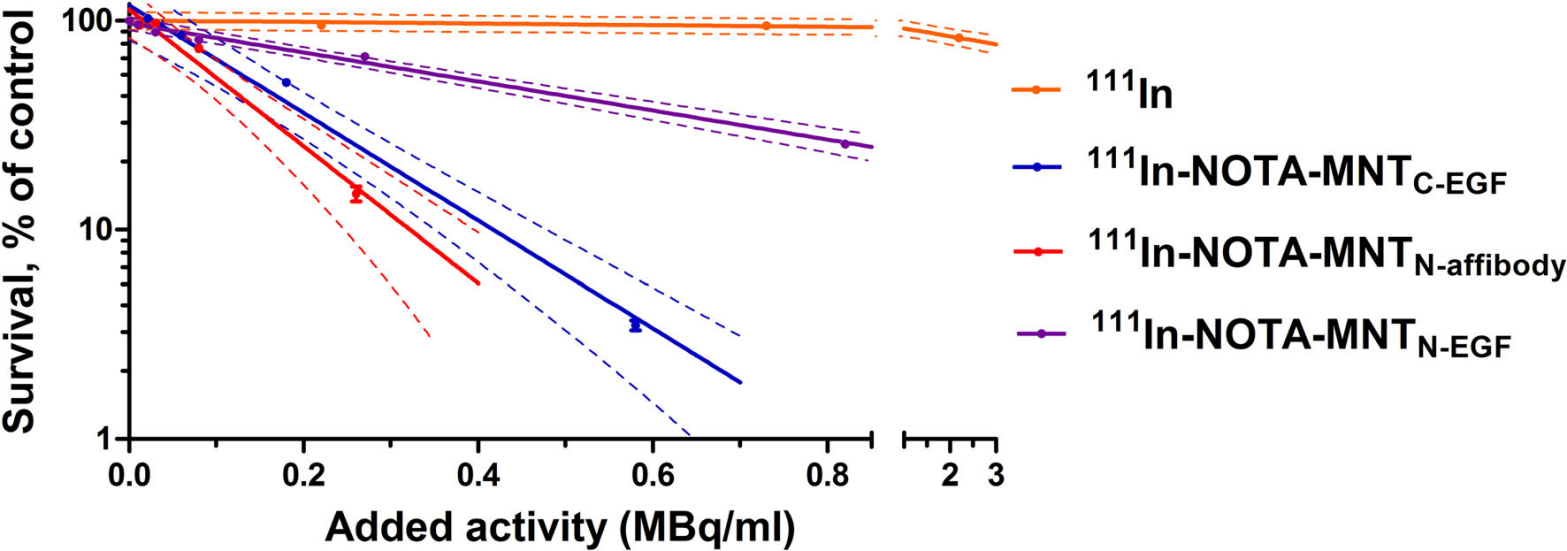
Cytotoxicity of ^111^In delivered by EGFR-targeted MNT. A431 cells were exposed for 48 h to various activity levels of ^111^In-NOTA-MNT or control ^111^In. Cells were seeded for a colony-forming assay at a density of 2,000 cells flask^−1^. After 8 d, the colonies were stained and counted. Solid lines represent data fitted to a mono-exponential model. Error bars are SEM (n = 3–6). Due to small values, in some cases, error bars merge with the symbols. Dotted lines represent 95% confidence intervals for each MNT and control ^111^In.

We demonstrated that the transfer and replacement of the ligand module did not affect to the functional activity of the remaining modules, and new transporters are able to reach the target compartment — the cell nucleus. On this basis, it seemed important to evaluate the cytotoxic effect of Auger electron emitter ^111^In, transported by new MNT to the nuclei of target cells. We used unbound ^111^In as a control. **Figure 8** shows clonogenic survival plots of A431 cells after 48 h incubation with serial dilutions of ^111^In-NOTA-MNT_C-EGF_, ^111^In-NOTA-MNT_N-affibody_, ^111^In-NOTA-MNT_N-EGF_ (0–6.5 MBq ml^−1^) and free ^111^In (0–20 MBq ml^−1^). The cytotoxicity of ^111^In delivered by any ^111^In-NOTA-MNT far exceeds that of control ^111^In. The ^111^In-NOTA-MNT_C-EGF_ and ^111^In-NOTA-MNT_N-affibody_ have similar cytotoxicity. The slopes of the curves for ^111^In-NOTA-MNT_C-EGF_ and ^111^In-NOTA-MNT_N-affibody_ differ significantly from the slopes of the curves for control ^111^In and ^111^In-NOTA-MNT_N-EGF_ according to the Tukey multiple comparison test (P < 0.05). The ^111^In activity levels of ^111^In-NOTA-MNT_C-EGF_ and ^111^In-NOTA-MNT_N-affibody_ that reduced clonogenic efficiency to 37% (A_37_) of the control were 0.2± 0.05 MBq ml^−1^ and 0.15± 0.04 MBq ml^−1^, respectively. In contrast, that was 0.6 MBq ml^−1^ ± 0.05 for ^111^In-NOTA-MNT_N-EGF_ A_37_ and 11.00 ± 2.00 MBq ml^−1^ for free ^111^In.

**Figure 9:**
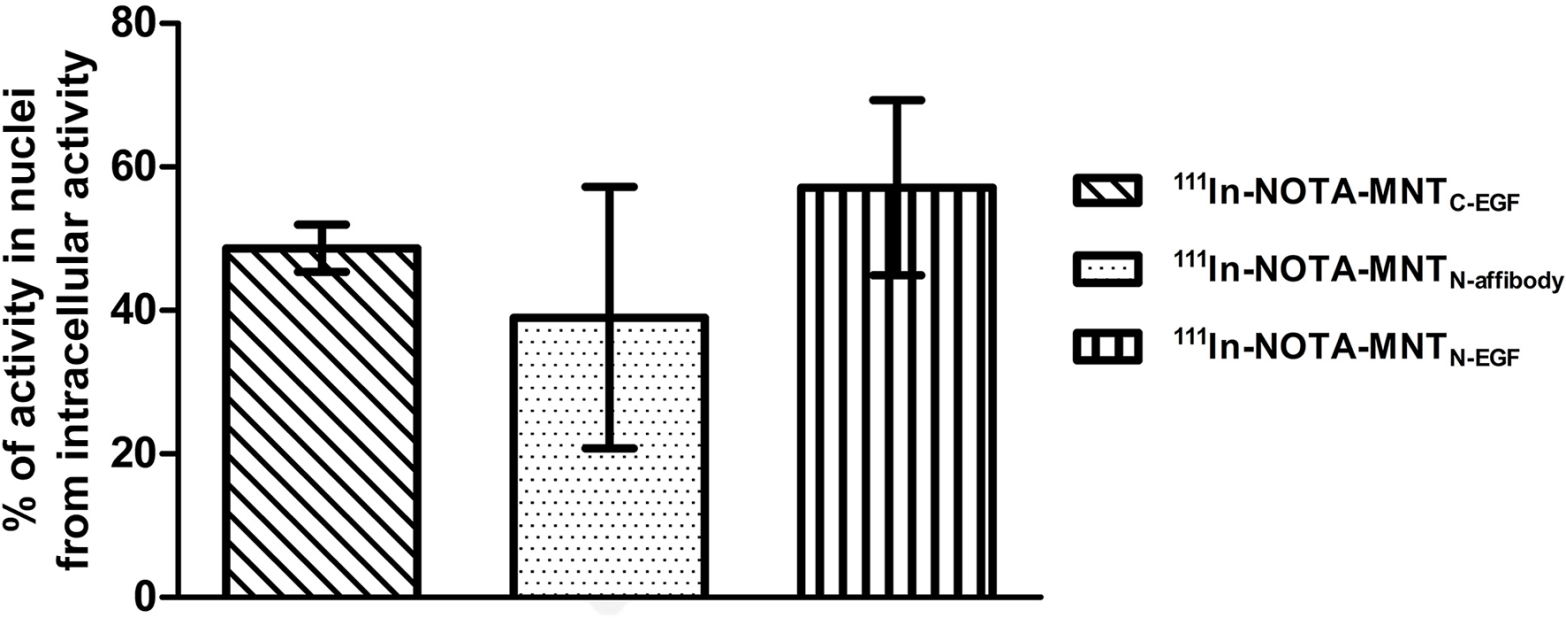
Intranuclear accumulation efficiency of ^111^In-NOTA-MNT. A431 cells were incubated with ^111^In-NOTA-MNT (0.6 MBq ml^−1^; 1.9 μg ml^−1^) for 2 h. Percentage of intranuclear accumulated activity relative to whole intracellular accumulated activity. Error bars are SEM (n = 3).

As ^111^In Auger electrons have extremely short ranges, their cytotoxicity was highest when they were localized in the nucleus (Li et al., 2015; Slastnikova et al., 2017a). Thus, we measured the ability of ^111^In-NOTA-MNT to accumulate in the nuclei. As shown in **Figure 9**, ^111^In was delivered to A431 nuclei by all EGFR-targeted MNT. The purity of the nuclear fractions was validated by western blot (**Supplements 2**).

The percentages of total intracellular radioactivity in the nuclei slightly differed between the various ^111^In-NOTA-MNT. For ^111^In-NOTA-MNT_N-affibody_, it was 39 ± 18%, for ^111^In-NOTA-MNT_C-EGF_ it was 49 ± 3%, and for ^111^In-NOTA-MNT_N-EGF_ it was 57 ± 12% (**Figure 9**). The significance of the difference was evaluated using the Mann–Whitney U-test. No statistically significant difference between the MNT was found.

**Figure 10:**
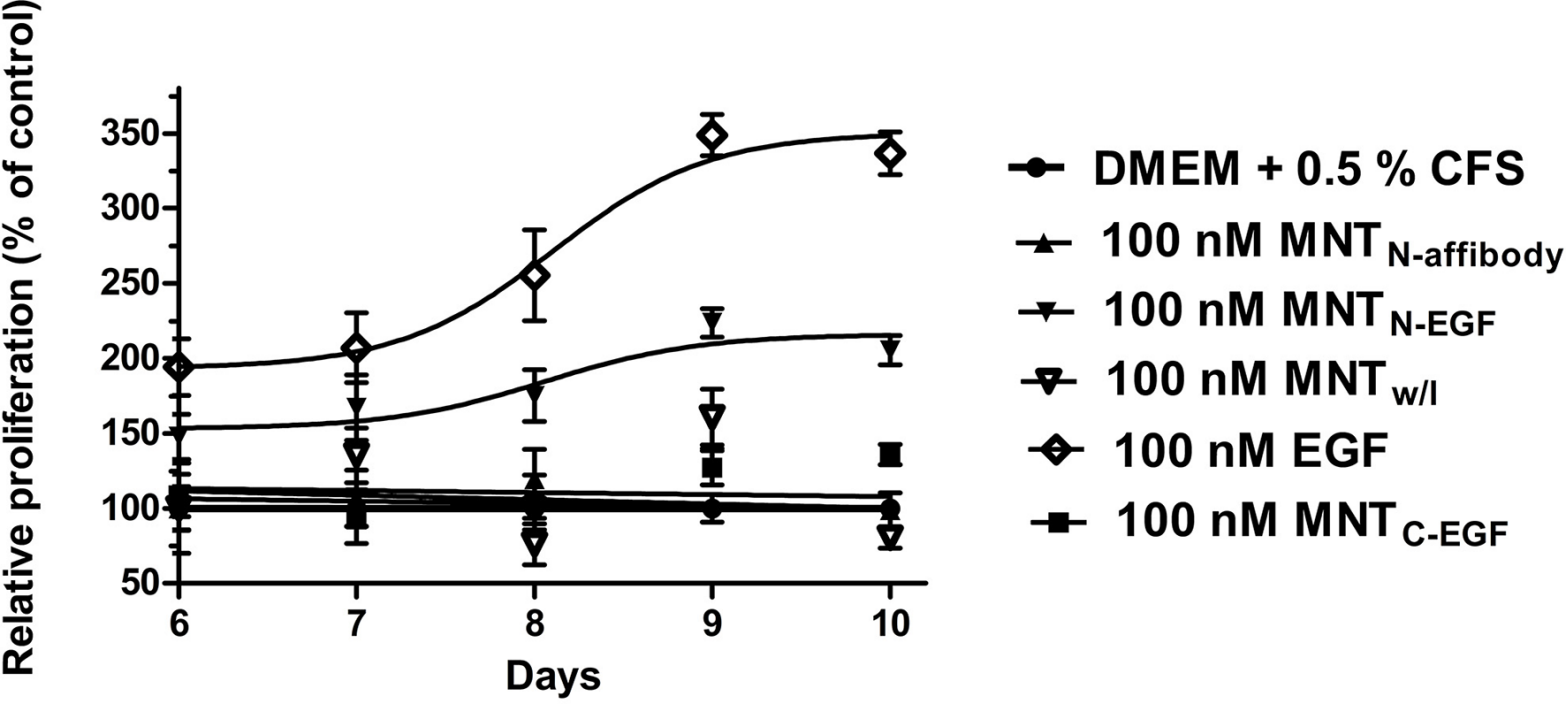
Effects of MNT on cell proliferation. MCF-7 cells were exposed for 5–9 d to 100 nM MNT or free EGF in medium with 0.5% serum. MNT and EGF were added 1 d after seeding. Cell proliferation was analyzed every 24 h by MTT assay starting at day 6. Proliferation values for untreated cells were set as 100% for each day. Other values were calculated as relative cell proliferation vs. corresponding values for untreated cells. Data points are represented as means ± standard deviation (SD) (n = 4).

We investigated the effects of MNT on cell proliferation. It was previously shown that several cell lines, like A431 and MDA-MB-468 are atypical (Kawamoto et al., 1983; Armstrong et al., 1994; Bromberg et al., 1998). They have a feature that distinguishes them from most other EGFR-expressing cancer cell lines. For these cell lines activation of EGFR leads to inhibition of the tumor cell proliferation, whereas for other types of tumor cells, such as MCF-7, activation of EGFR, on the contrary, leads to the induction of cell growth. We used the cell line, that does not have the feature in order to examine the ability of the MNT to induce cell proliferation relative to the control free EGF. We showed that the growth rate of MCF-7 cancer cell line greatly increased after a few days incubation with EGF and MNT_N-EGF_ compared to control cells. Therefore, these agents possessed pro-oncogenic effect. On the other hand, incubation with MNT_N-affibody_ and MNT_C-EGF_ had no statistically significant impact on MCF-7 proliferation (**Figure 10**).

## Discussion

EGFRs are highly convenient for the targeted delivery of various drugs into cancer cells. A number of drug delivery vehicles were produced for this purpose and evaluated *in vitro* and *in vivo* [(Chen et al., 2002; Lu et al., 2005; Liu et al., 2010; Song et al., 2016; Slastnikova et al., 2017b; Zahaf et al., 2017; Rosenkranz et al., 2018), etc.]. The main areas of EGFR-targeted drug therapy include: (1) development of drugs based on anti-EGFR antibodies binding to the extracellular EGFR domain, preventing ligand binding, and interrupting signal cascades (Herbst, 2004; Friedman and Stahl, 2009; Scott et al., 2012); (2) tyrosine kinase inhibitors binding to the intracellular EGFR domain and inhibiting the downstream effects of EGFR ligand binding (Herbst, 2004; Dassonville et al., 2007; Gazdar, 2009); and (3) delivery of drugs to cancer cells by fusion constructs containing EGF (Scott et al., 2012). However, several factors reduce EGFR-dependent treatment efficacy.

Most preclinical studies are conducted on xenograft models using immunodeficient mice (Cai et al., 2007; Eiblmaier et al., 2008; Liu et al., 2010). The relative efficacies of drugs targeting EGFR and tested on xenograft models could significantly decrease in the transition to clinical trials (Troiani et al., 2008; Day et al., 2015). Antibodies often have low interspecies cross-reactivity. Poor translation of results from xenograft to clinical trials may be especially severe for monoclonal anti-EGFR antibodies (Weller et al., 1987; Burgess, 2008).

Anti-hEGFR antibodies bind to xenograft cells overexpressing hEGFR. When the drug is administered to humans, however, most of the antibodies bind to liver cells resulting in the decreased proportion of antibodies reaching the tumor cells (Burgess, 2008). Consequently, drug efficacy is compromised, the incidence and severity of side effects dramatically increase, and therapy is adversely affected. Moreover, drug efficacy may be relatively overstated in immunodeficient animals with xenografts (Frese and Tuveson, 2007; Cekanova and Rathore, 2014). Thus, there is a growing risk that newly developed drugs may fail the clinical trial stage (Sharpless and Depinho, 2006; Ocana et al., 2010). Attempts have been made to overcome these limitations such as the creation of the Cetuximab analogue anti-mouse EGFR mAb 7A7 (Garrido et al., 2004). In contrast, efforts have failed in the development of simple models for preclinical studies of drugs exhibiting the same properties as human analogues such as 7A7 (He et al., 2018).

An alternative remedial approach is to develop a drug that binds mouse and human EGFR equally well. It would facilitate the accurate assessment of drug pharmacokinetics (Haeri and Osouli, 2017). The hEGFs have high binding affinities for hEGFR and mEGFR (Groenen et al., 1994; Elleman et al., 2001). Thus, the use of hEGFR as a ligand module would improve and harmonize therapeutic agent biodistribution in preclinical and clinical studies.

However, upon its interaction with EGFR on tumor cells, EGF induces receptor dimerization and autophosphorylation which activate several downstream kinase cascades. This leads to complex of prooncogenic effects such as cancer cell proliferation, migration, survival, DNA-double strand break repair, hypoxia, mediates radioresistance and inhibits apoptosis (Rodemann et al., 2007; Bussink et al., 2008). Hence, we selected affibody (Z_EGFR_:1907) as a ligand for MNT.

The affibody has shown strong binding affinity to EGFR with good specificity (Qi et al., 2012); it is significantly smaller (~ 7 kDa) (Orlova et al., 2007: Tolmachev et al., 2009), compared to multidomain antibodies (~ 150 kDa), ScFv (~ 28 kDa) and single-domain antibodies - nanobodies (~ 15 kDa) (Slastnikova et al., 2018). It has equal affinity for both mEGFR and hEGFR (Friedman et al., 2008; Qi et al., 2012) and does not induce receptor autophosphorylation and cell proliferation (Beuttler et al., 2009; Nordberg et al., 2010; Ekerljung et al., 2012). In addition, affibody molecules tolerate modifications and conjugations with retained high-target affinity, and the absence of disulfide bridges in the structure make them convenient to be used as a part of complex molecules (Friedman et al., 2008).Therefore, the affibody can be successfully used to deliver cytotoxic agents to the nuclei of cancer cells with EGFR expression.

Although the affibody on its own does not induce receptor autophosphorylation and cell proliferation, it seemed important to verify this for MNT_N-affibody_. MNT with EGF at the *N*- and *C*-termini and free EGF were used as controls. We investigated the effects of MNT on cell proliferation on the non-atypical cell model MCF-7, for which activation of EGFR leads to mitogenic effect. We used A431 cell line for the other experiments, since the results for the previously described MNT_C-EGF_ were obtained on this cell line (Gilyazova et al., 2006; Rosenkranz et al., 2008; Slastnikova et al., 2012a; Koumarianou et al., 2014; Slastnikova et al., 2017b; Rosenkranz et al., 2018), however for A431 activation of EGFR leads to inhibition of the tumor cell proliferation (Kawamoto et al., 1983; Bromberg et al., 1998). We showed that MNT_N-affibody_ did not affect cell growth (**Figure 10**), that was consistent with the properties of this affibody (Friedman et al., 2008; Beuttler et al., 2009; Ekerljung et al., 2012). MNT_C-EGF_ and MNT_N-EGF_ presented with different effects on MCF-7 proliferation depending on the position of the EGF-ligand module. Similar effects were reported for other EGF-containing chimeric constructs (Kim et al., 2015). МNТ_N-EGF_ may interact with EGFR like an epigen ligand whose *C*-terminal sequence is long compared with that of EGF. The epigen initiates EGFR dimerization which is relatively unstable but induces cell proliferation (Kochupurakkal et al., 2005; Freed et al., 2017). Nevertheless, this comparison may be inaccurate as we did not study the steric mechanisms of MNT interaction with EGFR. Here, we focused on the characteristics of MNT that pertain to the development of targeted drug delivery platforms.

All of the MNT examined here specifically bound at similar K_d_ to EGFR and were then internalized by receptor-specific mechanism (**Figures 3**, **5**, and **6**). The MNT-receptor complex underwent endocytosis and the MNT was enclosed in the endosome. The endosomolytic module was active at pH ~5.5 according to the liposome-based experimental model (**Figure 4**). This pH corresponds to that previously obtained for MNT_C-EGF_ (Gilyazova et al., 2006).

All MNT specifically bound to the α/β importin heterodimer responsible for nuclear import because of the activity of the NLS module. MNT_N-affibody_ and MNT_C-EGF_ had similar dissociation constants with the α/β importin heterodimer whereas that of MNT_N-EGF_ was comparatively lower (**Figure 7**). We suggested that this may be due to steric features for these molecules. We also showed that the signal from all EGF-binding MNT molecules is detected inside target cell nuclei (**Figures 6** and **9**) and it changes over time (from 7 to 48 h incubation), while for MNT_w/l_ it remains equally low (**Figure 6C**).

In this study, we selected the Auger electron emitter ^111^In as the cytotoxic agent, which has a local damaging effect. The range of its Auger electrons is several tens of nanometers. Thus, ^111^In is suitable for nucleus-targeted therapy (Kassis, 2004). When ^111^In was delivered into the nuclei of target cancer cells *via* MNT, its cytotoxicity was markedly greater than that of the free ^111^In used as a control. These data correspond to those previously reported (Slastnikova et al., 2017b; Rosenkranz et al., 2018). However, ^111^In-NOTA-MNT_N-affibody_ and ^111^In-NOTA-MNT_C-EGF_ demonstrates higher cytotoxicity than ^111^In-NOTA-MNT_N-EGF_ (**Figure 8**). The lowest cytotoxicity of ^111^In-NOTA-MNT_N-EGF_ may be explained by the oppositely directed effect of this type of MNT on the target cells, because of EGF in this version of MNT apparently may activate EGFR and induce signaling cascades, which leads to cell survival cell cycle progression, inhibition of apoptosis, etc. (Rodemann et al., 2007; Bussink et al., 2008, Sigismund et al., 2018). This is evidenced, in particular, by the stimulation of cell proliferation by this MNT.

Based on the results of the present study, we consider MNT_N-affibody_ as a promising vehicle for anticancer agents for targeted therapy. It successfully delivers ^111^In inside target cell nuclei and result in cancer cell death.

The obtained data provide the basis for future experiments both *in vitro* and *in vivo*. In vitro study of further other possible types of cellular response to interaction with MNT, such as the level of double-strand DNA breaks (DSB) and apoptosis will be valuable. In vivo the effects of the new MNT should be evaluated. Prolonged intratumoral retention of ^111^In-NOTA-MNT_C-EGF_ with t_1/2_ = 4.1 ± 0.5 days as well as significant dose-dependent tumor growth delay (up to 90% growth inhibition) after intratumoral administration of ^111^In-NOTA-MNT_C-EGF_ (Rosenkranz et al., 2018) permits us to suggest the similar *in vivo* characteristics for this new MNT. Further modifications of the MNT aimed at reduction of its immunogenicity can provide a basis for efficient systemic use of the regarded MNT.

## Conclusion

A new MNT with an affibody ligand was designed and characterized. MNT_N-affibody_ specifically binds to EGFR. MNT_N-affibody_ accumulated inside the target cells wherein it was transported into the nuclei. MNT_N-affibody_ delivered the Auger electron emitter ^111^In into the target cell nuclei. The cytotoxicity of ^111^In delivered by MNT_N-affibody_ was far greater than that of free ^111^In. Unlike EGF and MNT_N-EGF_, however, it does not induce target cell proliferation. Taken together, these results suggest that MNT_N-affibody_ is a promising targeted drug delivery therapy against cancers characterized by EGFR overexpression.

## Data Availability Statement

All datasets generated for this study are included in the article/**Supplementary Material**.

## Author Contributions

AS, AU, and AR designed and evaluated the study. AU constructed plasmids of the new MNT by genetic engineering, and TL and TK made the proteins purification. TK, TS, AR, and YK carried out experiments to evaluate the activity of functional modules of the MNT and cell culture experiments. AS, AU, TS, AR, TL, YK, GG, and TK contributed to the discussion and interpretation of the results. TK took the lead in writing the manuscript. All authors agreed to be accountable for all aspects of the work and read and approved the final manuscript.

## Funding

The research was supported by RSF (project No. 17-14-01304).

## Conflict of Interest

The authors declare that the research was conducted in the absence of any commercial or financial relationships that could be construed as a potential conflict of interest.

## Supplementary Material

Supplement 1 Amino acid sequences of the MNT. Individual modules are highlighted in different colors. His-Tag is highlighted in red.

Supplement 2 Purity of isolated nuclei verified by enhanced chemiluminescence (ECL) western blot. (**α**) Bands from PageRuler™ Prestained Protein Ladder. (**β**) Cytoplasmic fraction. (**γ**) Nuclear fraction. NBS-1 antibody recognizes PageRuler™ Prestained band at 95 kDa which represents NBS-1.
