# Supplementary figures and images for: Targeted Delivery of 111In Into the Nuclei of EGFR Overexpressing Cells via Modular Nanotransporters With Anti-EGFR Affibody
Source: Front Pharmacol. 2020 Mar 4;11:176. doi: 10.3389/fphar.2020.00176 (PMC7064642; doi:10.3389/fphar.2020.00176)

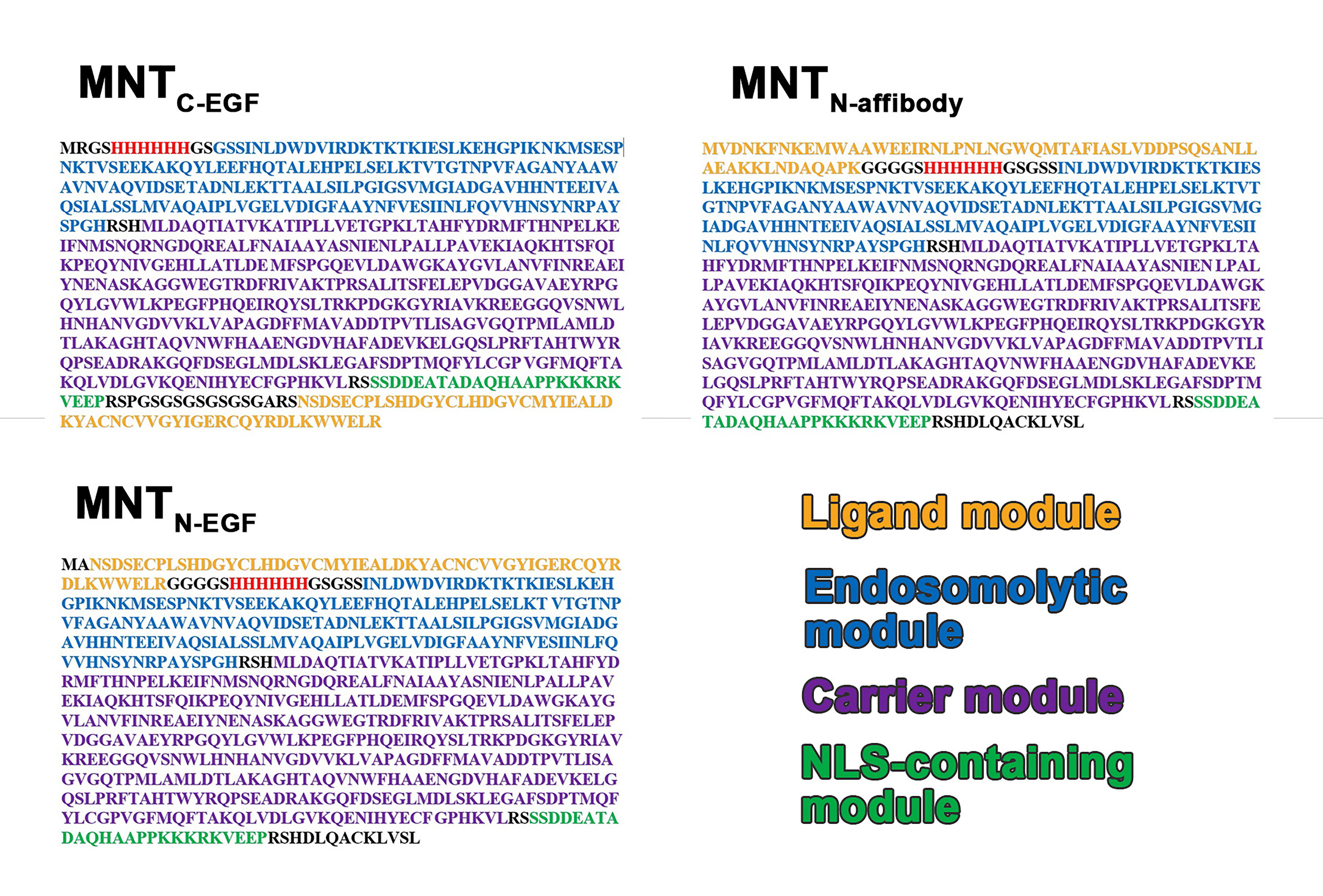

Supplement: Supplement 1 — Amino acid sequences of the MNT. Individual modules are highlighted in different colors. His-Tag is highlighted in red. [file Image_1.jpg]

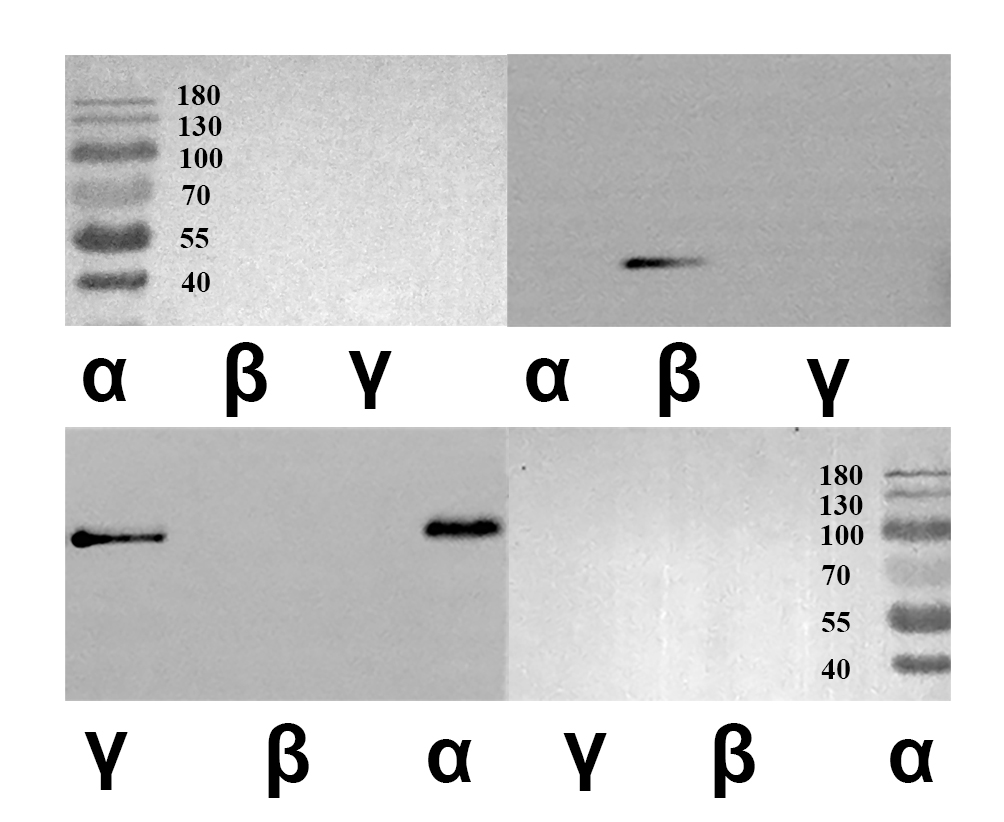

Supplement: Supplement 2 — Purity of isolated nuclei verified by enhanced chemiluminescence (ECL) western blot. (α) Bands from PageRuler™ Prestained Protein Ladder. (β) Cytoplasmic fraction. (γ) Nuclear fraction. NBS-1 antibody recognizes PageRuler™ Prestained band at 95 kDa which represents NBS-1. [file Image_2.jpg]
